# Medication prescribing errors among hospitalized pediatric patients at Nekemte Referral Hospital, western Ethiopia: cross-sectional study

**DOI:** 10.1186/s13104-019-4455-1

**Published:** 2019-07-16

**Authors:** Ginenus Fekadu, Eba Abdisa, Korinan Fanta

**Affiliations:** 1 Clinical Pharmacy Unit, Department of Pharmacy, Institute of Health Sciences, Wollega University, Nekemte, Ethiopia; 2 Department of Psychiatry, School of Nursing and Midwifery, Institute of Health Sciences, Wollega University, Nekemte, Ethiopia; 3 Clinical Pharmacy Unit, School of Pharmacy, Institute of Health, Jimma University, Jimma, Ethiopia

**Keywords:** Prescribing error, Pediatrics, Medications, Nekemte, Ethiopia

## Abstract

**Objective:**

Incidence and clinical outcomes of medication prescribing errors are common and potentially more harmful in the pediatric population than in the adult population. Hence, this study was aimed to assess the prevalence and types of medication prescribing errors in the pediatric wards of Nekemte Referral Hospital (NRH).

**Results:**

Of 384 pediatric patients included in the study, 241 (63%) were males and 116 (30.21%) of them were aged between 1–3 years. About 241 (62.76%) of the patients were treated based on empirical diagnosis and only 10 (2.60%) pediatrics had co-morbid disease. The most category of medication prescribing error was dosing error 251 (48.6%) followed by incorrect drug selection 98 (19.0%). Being critically ill (AOR = 5.31, 95% CI = 1.80–12.31, p = 0.003), route of administration via IV (AOR = 3.98, 95% CI = 1.85–11.15, p = 0.011) and via IV + IM route (AOR = 2.22, 95% CI = 1.05–9.25, p = 0.045) as well as 4–6 medications per patient (AOR = 3.10, 95% CI = 3.43–12.42, p = 0.012) and > 6 medications per patient (AOR = 7.23, 95% CI = 3.91–21.45, p < 0.001) were independent predictors of medication prescribing errors. Antibiotics were the most common classes of drugs responsible for prescribing errors.

## Introduction

Prescription is an instruction written from a prescriber to a drug dispenser [1, 2]. It is considered as document that should be written clearly and accurately as well as should indicate precisely what should be given to patients [3]. There are two main categories of prescribing error; these are omission error (missing essential information) and commission error (addition of wrong information) [2]. Despite, medication prescribing error is a preventable event; its incidence and clinical effect were common among pediatric population. Compared to adults’ pediatric population are sensitive to harmful consequences of medication prescribing error due to different factors such as rapid physiological change, pharmacokinetic variations, organ maturity, variation in age and weight [3–9].

High medication error rates with significant consequences occur in intensive care units but errors could be minimized with intensive follow-up and appropriate monitoring of the medications [10]. Reviewing orders and prescriptions by pharmacists is critical step for detecting errors and preventing adverse impacts on patients [11, 12]. Adherence to basic prescription writing order delivers appropriate information to dispensers by delivering appropriate information for the treatment of patients. In contrary to this, failure to adhere to standard prescription writing order can cause drug–drug interaction, toxicity, exacerbation of the illness, and poor treatment outcomes that can lead to high economic crisis and lose of the patients’ life [2]. Therefore, auditing prescription and assessing medication prescription errors are important to give appropriate feedback and to ensure rational prescribing among prescribers [2, 5].

Data regarding pediatrics prescribing error in Ethiopian health setups is almost absent [8]. Therefore, this study was aimed to assess the prevalence and types of prescribing errors at pediatric wards of NRH.

## Main text

### Patients and methods

#### Study setting and study design

Institutional based cross sectional study was conducted at NRH from February to April 2017, Nekemte town, western Ethiopia.

#### Eligibility criteria

Pediatric patients admitted to NRH who had medication prescription were included irrespective of treatment outcome. While pediatric patients who were self-discharged and those > 12 years of age were excluded from the study.

#### Sample size and sampling technique

Single population proportion formula was used to calculate sample size using the level of significance taken as 95%, (Zα = 1.96), error of margin = 5% and P was assumed to be 50%. Accordingly, the minimum sample size was fixed to be 384.

$${\text{n}} = \frac{{\frac{{(Z\alpha )^{2} }}{2}{\text{p(1}} - {\text{p)}}}}{{{\text{d}}^{{\text{2}}} }}$$

where, p = extent of adherence of prescribers to non-prescription error. Z = critical value at 95% CI of certainty (1.96). d = the margin of error (5%). n = the required sample size.

Therefore, the sample size was

$${\text{n}} = \frac{{(1.96)^{2} \left( {0.5} \right)\left( {1 - 0.5} \right)}}{{\left( {0.05} \right)^{2} }}\quad {\text{n}} = 3 8 4$$

Random sampling technique was employed to select the study participants.

#### Data collection process and quality assurance

Semi-structured data collection tool was utilized to collect necessary data from patient cards and medication charts. Data quality was assured by careful selection and collection of complete data. The clarity and completeness of the data collection tool was checked before the actual data collection started. A 5% sample pretest was performed on randomly selected patients at Gimbi General Hospital before the beginning of the study.

#### Data processing and analysis

The collected data was analyzed using SPSS version 20. Prescribing errors were identified by comparing with “National standard treatment guideline” and “Pocket book of pediatric hospital care in Ethiopia” [13–15]. Independent predictors of prescribing errors were analyzed using the logistic regression model by estimating the odds ratio (OR) and 95% CI for each covariates. Confidence interval which doesn’t contain 1 and predictor variables with probability value less than 0.05 was considered statistically significant.

### Results

#### Socio-demographic characteristics of patients

Of a total of 384 pediatric patients included in the study, 241 (63%) were males while the rest 143 (37%) were females. About one-third of study participants (30.21%) were between 1-3 years of age and infants consisted of 112 (29.17%). Regarding the weight, one-third (33.33%) of the patients weigh between 5.1 and 10 kg (Table 1).

**Table 1:** Socio demographic characteristics of pediatric patients admitted to pediatric wards of NRH, February 1 to April 30, 2017

Socio demographic characteristics	Frequency and percentage		
	Male	Female	Total
Age			
Infant (1–12 months)	56 (14.58%)	56 (14.58%)	112 (29.17%)
Toddler (1–3 years)	80 (20.83%)	36 (9.38%)	116 (30.21)
Preschool (3–5 years)	28 (7.29%)	36 (9.38)	64 (16.67%)
School age (5–10 years)	51 (13.28%)	10 (2.60%)	61 (15.88%)
Adolescent (10–12 years)	26 (6.77%)	5 (1.30%)	31 (8.07%)
Total	241 (62.75%)	143 (37.24%)	384 (100%)
Body weight (kg)			
≤ 5.0	20 (5.21%)	11 (2.86%)	31 (8.07%)
5.1–10	76 (19.79%)	52 (13.54%)	128 (33.33%)
10.1–15	60 (15.635%)	21 (5.47%)	81 (21.1%)
15.1–20	20 (5.21%)	16 (4.16%)	36 (9.37%)
> 20	20 (5.21%)	6 (1.56%)	26 (6.77%)
Not recorded	45 (11.77%)	37 (9.64%)	82 (21.36)
Total	241 (62.77%)	143 (37.24%)	384 (100%)

#### Diagnosis approaches used

Out of 384 hospitalized pediatric patients, 241(62.76%) were treated based on empirical diagnosis and 5(1.30%) were treated based on laboratory tests/kinetic diagnosis. The remaining, 138(35.94%) were treated based on both empirical and kinetic diagnosis.

#### Clinical characteristics of the patients and medication prescribing errors

During the study period, 10 (2.60%) pediatrics had co-morbid disease and about half of the patients (50.78%) were severely ill. Out of a total study participants, 261 (67.97%) of them had suffered from medication prescribing errors. The most category of medication prescribing error was dosing error 251 (48.6%), followed by incorrect drug selection comprising 98 (19.0%). Under dosing comprises of 198 (38.37%) of the dosing errors. The medications which were not fully prescribed accounts 36 (9.38%) while prescriptions with incorrect instructions comprises 10 (2.60%). The sampled prescriptions contain a total of 1596 drugs. The average number of drugs per prescription was found to be 4.16 (Table 2).

**Table 2:** Clinical characteristics, prescribed medication and types of medication errors among pediatric patients admitted to pediatric wards of NRH, February 1, 2015–April 30, 2017

Clinical characteristics	Frequency	Percentage (%)	
Had co- morbid disease	10	2.60	
Severity of the disease (condition of the patient)			
Acute	51	13.28	
Severe	195	50.78	
Critical	138	35.94	
Previous medical history	61	15.89	
Previous medication history	51	13.28	
Previous hospital admission	41	10.68	
Pediatrics with new cases	323	84.11	
Number of medications per patient			
1–3	112	29.17	
4–6	204	53.12	
> 6	68	17.71	

Types of medication errors	Frequency	Percentage (%)	
Incorrect drug strength			
Overdose	53	10.27	
Under dose	198	38.37	
Incorrect drug dosage	98	19.0	
Drugs not fully prescribed	87	16.86	
Incorrect drug selection	48	9.3	
Drugs with incorrect instruction	32	6.20	

Prescribed drugs	Route of administration	Frequency	Percentage (%)
Ceftriaxone	IV only	257	22.13
Paracetamol	PO + Anal	205	17.72
Gentamicin	IM + IV	189	16.34
Cephalexin	PO only	118	10.19
Amoxicillin	PO only	87	7.52
Cloxacillin	PO only	72	6.22
Chloramphenicol	IM + IV + PO	67	5.79
Salbutamol syrup	PO only	61	5.27
Ampicillin	IM + IV + PO	51	4.41
Hydrocortisone	IV only	51	4.41
Total		1157	100

*IV* intravenous; *IM* intramuscular; *PO* orally

#### Predictors of prescribing errors

After correction for other variables on multivariable logistic regression analysis severity of the disease, route of drug administration and number of medications per patient were strongly associated with medication errors at p < 0.05. Critically ill patients were almost 5 times more likely to have medication errors (ME) than patients who were acutely ill (AOR = 5.31, 95% CI 1.80–12.31, p = 0.003). Patients who had administered medication via IV route were 4 times more to experience MEs compared to patients who had administered per oral (AOR = 3.98, 95% CI 1.85–11.15, p = 0.011). Patients who had administered medication via IV + IM route were about 2 times more likely to experience MEs than patients who had administered per oral (AOR = 2.22, 95% CI 1.05–9.25, p = 0.045). Patients who had prescribed 4–6 medications concomitantly were 3 times more likely to have MEs than patients who had used 1–3 medications (AOR = 3.10, 95% CI 3.43–12.42, p = 0.012), in the same way patients who had prescribed > 6 medications concomitantly were about 7 times more likely to have MEs than patients who had used 1–3 medications (AOR = 7.23, 95% CI 3.91–21.45, p < 0.001) (Table 3).

**Table 3:** Multivariable analysis of factors associated with MEs among hospitalized pediatrics in pediatric wards of NRH, February 1, 2015–April 30, 2017

Variables	Category	COR (95% CI)	P value	AOR (95% CI)	P value
Severity of the disease	Acute	1.00	–	1.00	–
	Severe	2.27 (1.63–6.56)	0.002	1.21(0.52–4.24)	0.087
	Critical	6.12 (2.40–11.81)	0.001	5.31(1.80–12.31)	0.003
Routes of administration	PO	1.00	–	1.00	–
	IV	4.46 (2.53–12.57)	0.000	3.98 (1.85–11.15)	0.011
	IV + IM	2.36 (1.21–10.47)	0.002	2.22 (1.05–9.25)	0.045
	IV + PO	2.10 (0.98–4.10)	0.070	0.83 (0.55–2.55)	0.978
	PO + anal	1.98 (0.29–4.57)	0.084	1.34 (0.20–3.80)	0.531
	Others	0.65 (0.89–8.14)	0.655	0.35 (0.67–7.65)	0.884
Number of medications per patient	1–3	1.00	–	1.00	–
	4–6	3.14 (4.63–11.67)	0.000	3.10 (3.43–12.42)	0.012
	> 6	7.65 (4.43–18.67)	0.0001	7.23 (3.91–21.45)	0.000

*AOR* adjusted odds ratio; *COR* crude odds ratio; *IV* intravenous; *IM* intramuscular; *PO* oral

### Discussion

This study was carried out with the aim of assessing the prevalence of medication prescribing errors in a resource limited setting particularly in Ethiopia. This study showed that 67.97% of pediatric patients had been exposed to at least one ME. Slightly comparable findings were reported from Dessie referral hospital [8], Nekemte referral hospital [15] and Jimma University specialized hospital [16] which reported that, 58.07%, 75.1% and 52.5% of the patients experienced at least one medication error, respectively. However, our finding was almost more than double compared to the study conducted in USA, where only 28.6% of patients had at least one ME [17].This difference could be due to differences in definitions of errors, methods used to detect errors, availability of facilities for patient care and role of pharmacist in the health care team. In current study, the sampled prescriptions contain a total of 1596 drugs. The average number of drugs per prescription paper was found to be 4.16. This was higher than the acceptable World Health Organization (WHO) ideal ranges (1.6–1.8) [18].

Pediatrics were prone to medication errors, predominantly because of the need for dosage calculations, which are individually based on the patient’s weight, age, body surface area and their condition [17]. Dosing errors that includes selecting incorrect drug strength in pediatrics might lead to toxicity. Thus checking the drug, the dose, patient identity and any other relevant information before administering medicine is mandatory [19].

According to the present study the most frequent MEs was dosing error 48.64%, which was in line with study done in Palestine were 40.0% of the medications were prescribed with one or more inappropriate doses [20]. However, our finding was higher compared to previous report from Nekemte referral hospital 23% [15], Dessie Referral Hospital 31.39% [8], Saudi Arabia 22.1% [21] and two studies done in USA, 28% and 28.4% [17, 22]. Additionally, it was unlike to study done in Jimma University specialized Hospital where the most common type of medication prescribing error was the wrong combination of drugs (25.7%) [16]. This discrepancy might be due to difference in completeness of data, hospital setup, medical condition of the patient and health care professionals’ experience.

In this study antibiotics were the most common classes of drugs responsible for prescribing errors; which was in line with study conducted in United Kingdom, Jimma University specialized Hospital and Palestine [16, 20, 23]. Antimicrobial agents can be prescribed empirically without awaiting definite identification of the causative agent [24]. It was a great issue that only 1.3% of prescribed antibiotics were prescribed depending on the results of culture and sensitivity. This is mostly due to budget constraint and facility deficit in resource limited settings including our study hospital where it is not feasible to do culture and sensitivity test for each and every patients. The microbiological test is almost performed in our set up only for research and investigation purpose as well as in some cases when resistance is suspected.

With regard to predictors of medication prescribing errors; severity of the disease, routes of administration and number of medication per patient were found to be independent predictors of medication prescribing errors. Parenteral route was more likely associated with prescribing errors. This was in contrast with study done at Dessie Hospital revealing that intravenous route was less likely to be associated with prescribing errors [8]. Additionally, study by Dedefo et al. showed that route of administration was not shown to be a predictor of MEs in multivariable analysis (p > 0.05) [15]. But our finding complies with study done in Saudi Arabia and Palestine where intravenous route (IV) has been reported as the most common causes of medication errors in children admitted to hospitals [20, 21]. This might be because parenteral route of administration is the most common route of drug administration for hospitalized pediatric patients.

Severity of the disease was one independent predictor of prescribing errors. As severity of the disease increases the number of medications used and errors also promptly increases. As the number and severity of disease increases, the number of medications required to treat conditions of the patient also increases. The present study showed that the number of medications used by the patient was significantly associated (p < 0.05) with MEs and it was one of the independent predictors of MEs. This was in-line with previous study done in NRH [15] reporting that the more medications a patient is consuming, the more likely for the occurrence of MEs.

### Conclusion

The study revealed that the prevalence of medication prescribing errors was high in pediatric wards of NRH. Severity of the disease, routes of administration and number of medication per patient were found to be independent predictors of medication prescribing errors. Antibiotics were the most common class of drugs involved in medication prescribing errors.

## Limitation of the study

Given the cross-sectional study design was employed, it was not possible to establish causal relationships due to the lack of a temporal link. Additionally, incomplete information from patient’s medical cards was also another issue. Finally, since the study included patients admitted to a single hospital, generalization of findings must be made cautiously.

## Data Availability

The datasets used and/or analyzed during the current study are available from the corresponding author on reasonable request.

## Abbreviations

FMHACA: Food, Medicine and Healthcare Administration and Control Authority
ME: Medication error
NRH: Nekemte Referral Hospital
